# Genomic Characterization of *Theileria luwenshuni* Strain Cheeloo

**DOI:** 10.1128/spectrum.00301-23

**Published:** 2023-06-01

**Authors:** Bai-Hui Wang, Li-Feng Du, Ming-Zhu Zhang, Luo-Yuan Xia, Cheng Li, Zhe-Tao Lin, Ning Wang, Wan-Ying Gao, Run-Ze Ye, Jin-Yue Liu, Xiao-Yu Han, Wen-Qiang Shi, Xiao-Yu Shi, Jia-Fu Jiang, Na Jia, Xiao-Ming Cui, Lin Zhao, Wu-Chun Cao

**Affiliations:** a Institute of EcoHealth, School of Public Health, Shandong University, Jinan, Shandong, People’s Republic of China; b State Key Laboratory of Pathogen and Biosecurity, Beijing Institute of Microbiology and Epidemiology, Beijing, People’s Republic of China; University of Huddersfield

**Keywords:** *Theileria luwenshuni* strain Cheeloo, whole-genome sequence, FISH, genomic characteristics, phylogenetic analysis

## Abstract

*Theileria*, a tick-borne intracellular protozoan, can cause infections of various livestock and wildlife around the world, posing a threat to veterinary health. Although more and more *Theileria* species have been identified, genomes have been available only from four *Theileria* species to date. Here, we assembled a whole genome of *Theileria luwenshuni*, an emerging *Theileria*, through next-generation sequencing of purified erythrocytes from the blood of a naturally infected goat. We designated it *T. luwenshuni* str. Cheeloo because its genome was assembled by the researchers at Cheeloo College of Medicine, Shandong University, China. The genome of *T. lunwenshuni* str. Cheeloo was the smallest in comparison with the other four *Theileria* species. *T. luwenshuni* str. Cheeloo possessed the fewest gene gains and gene family expansion. The protein count of each category was always comparable between *T. luwenshuni* str. Cheeloo and *T. orientalis* str. Shintoku in the Eukaryote Orthologs annotation, though there were remarkable differences in genome size. *T. luwenshuni* str. Cheeloo had lower counts than the other four *Theileria* species in most categories at level 3 of Gene Ontology annotation. Kyoto Encyclopedia of Genes and Genomes annotation revealed a loss of the c-Myb in *T. luwenshuni* str. Cheeloo. The infection rate of *T. luwenshuni* str. Cheeloo was up to 81.5% in a total of 54 goats from three flocks. The phylogenetic analyses based on both 18S rRNA and *cox1* genes indicated that *T. luwenshuni* had relatively low diversity. The first characterization of the *T. luwenshuni* genome will promote better understanding of the emerging *Theileria*.

**IMPORTANCE** *Theileria* has led to substantial economic losses in animal husbandry. Whole-genome sequencing data of the genus *Theileria* are currently limited, which has prohibited us from further understanding their molecular features. This work depicted whole-genome sequences of *T. luwenshuni* str. Cheeloo, an emerging *Theileria* species, and reported a high prevalence of *T. luwenshuni* str. Cheeloo infection in goats. The first assembly and characterization of *T. luwenshuni* genome will benefit exploring the infective and pathogenic mechanisms of the emerging *Theileria* to provide scientific basis for future control strategies of theileriosis.

## INTRODUCTION

*Theileria* is an obligate intracellular protozoan distributed around the world, and often causes disease in a variety of livestock and wildlife (1, 2), posing a substantial threat to veterinary health. Six *Theileria* species are known to infect cattle (3), among which *Theileria parva*, *Theileria orientalis*, and *Theileria annulata* are the most pathogenic and economically important (4–6). *Theileria equi* that can infect horses is a significant concern. The *Theileria* species is also capable of infecting wild ruminants, such as zebu cattle (7), African buffaloes (8), wild red deer, and sika deer (9). *Theileria lestoquardi* and *Theileria luwenshuni* are highly pathogenic, especially to goats, leading to economic losses in animal husbandry (10–13). In addition, several *Theileria* species emerged recently and might cause infection and disease in various livestock and wildlife in different regions of the world (14, 15), Although some *Theileria* species tend to be carried asymptomatically by hosts, several can result in diseases, and even death (16–22).

The species identification within the genus *Theileria* is usually based on the phylogenetic analysis of 18S rRNA gene (2, 23, 24). So far, only 12 genome sequences belonging to four *Theileria* species have been published and deposited in GenBank, including *T. orientalis*, *T. parva*, *T. annulata*, and *T. equi* (5, 6, 25, 26). The whole genomes of other *Theileria* species have not been available, which has prohibited us from understanding their evolutionary, genetic, and pathogenic characteristics. In a survey on tick-borne infection of goats, *T. luwenshuni* was detected in a flock of goats from Shandong Province of eastern China. We selected a blood sample positive for *T. luwenshuni* to get the genome sequence of the endoparasite using metagenomic sequencing. Considering *Theileria* is abundant in erythrocytes, in this study, we separated erythrocytes from the blood of the infected goat to enrich the parasite, and then assembled the whole genome of *T. luwenshuni* to characterize its genomic composition in comparison with genomes of other *Theileria* species, and to further evaluate the prevalence of the emerging *Theileria* species in hosts.

## RESULTS

### Identification of *T. luwenshuni* in goat blood.

**FIG 1:**
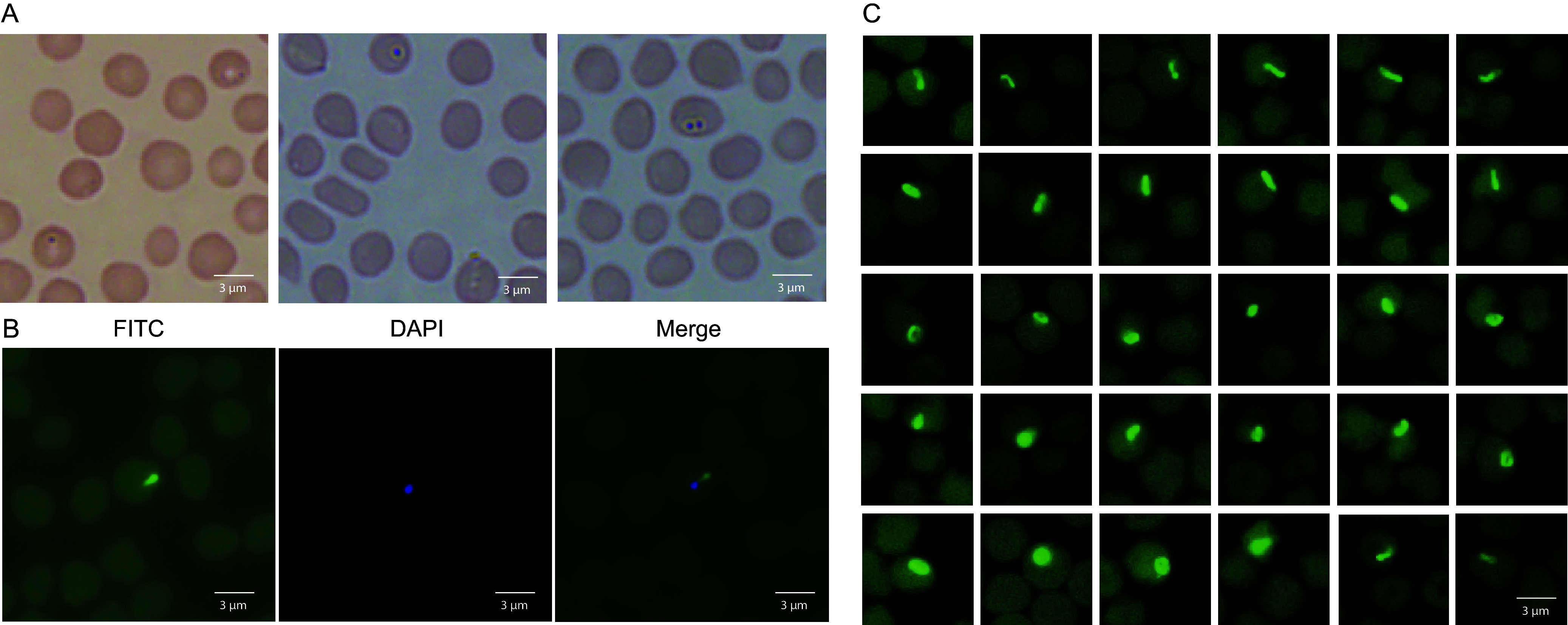
Morphology and size of *T. luwenshuni* str. Cheeloo inside erythrocytes. (A) Wright-Giemsa staining of goat blood smears (1,000×). (B) Fluorescence *in situ* hybridization (FISH) results under fluorescence microscope of *Theileria luwenshuni* str. Cheeloo. From left to right represent the fluorescein isothiocyanate (FITC) channel (fluorescence channel), the DAPI channel, and the merge channel. The blue fluorescence is the nucleus of the *Theileria* and the green strong fluorescence is the target *T. luwenshuni* str. Cheeloo. (C) FISH results showing different shapes and sizes of *T. luwenshuni* str. Cheeloo in erythrocytes.

In a survey on tick-borne infections in goats, *T. luwenshuni* was detected in blood samples of goats from Shandong Province of eastern China (see Fig. S1 in the supplemental material) by PCR using universal primers targeting the partial 18S rRNA gene (424 bp) of *Theileria* and *Babesia* (Table S1). BLAST analysis of the amplified fragment sequences revealed a 99.06% identity with the corresponding part of the *T. luwenshuni* HNQZ strain from goats in Hainan Province, China (GenBank accession no. MK685118.1). One or two *Theileria* could be visualized in each infected erythrocyte on the blood smear prepared from infected goats by Wright-Giemsa staining (Fig. 1A). To further confirm the presence of *T. luwenshuni*, we conducted a fluorescence *in situ* hybridization (FISH) assay using a specific probe designed according to 18S rRNA gene sequence and labeled with FAM, a green fluorescein. *T. luwenshuni* was observed with the nucleus stained by DAPI in the blood smears (Fig. 1B). They were polymorphic, including rod-shaped, pear-shaped, ovoid, or round. Their sizes were about 1.5 μm on average, ranging from 1 to 2 μm (Fig. 1C).

### Next-generation sequencing of blood from an infected goat.

A blood sample with high infection rate of erythrocytes from a naturally infected goat (No. 2) was chosen for next-generation sequencing. We first separated erythrocytes from 15 mL blood, and then lysed erythrocytes to enrich *T. luwenshuni* and primarily remove nucleic acid of the host goat. Total DNA was extracted from the supernatant of the lysed liquid, and DNA yields and purity were measured by automated electrophoresis. Then the sequencing library was prepared according to the whole-genome sequencing library preparation protocol (DNBSEQ). The metagenome sequencing resulted in over 39.3 million 150-bp Illumina reads from the sample. Despite primary removing of host DNA, 95.9% reads were mapped to the goat genome, and were discarded. The remaining reads were then *de novo* assembled into scaffolds using the SPAdes program (v3.15.3) with meta parameters. After assembly and binning, the *T. luwenshuni* genome with 7.61 Mb was discovered in assembly result, and named *Theileria luwenshuni* str. Cheeloo (GenBank accession no. GCA_029379255.1), because it was initially obtained by the researchers at Cheeloo College of Medicine, Shandong University, China.

### Genomic composition and phylogenetic position of *T. luwenshuni* str. Cheeloo.

**FIG 2:**
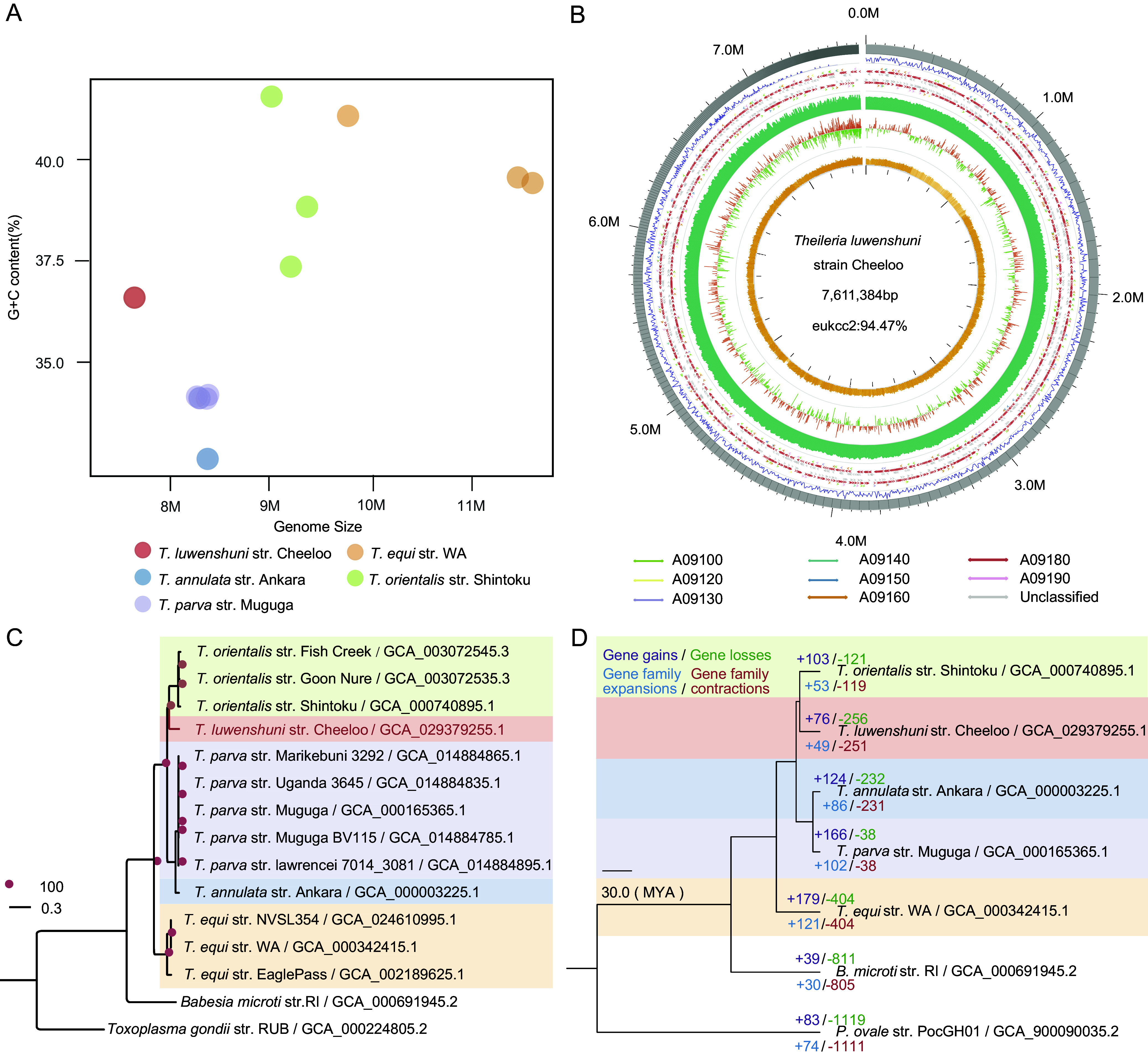
Genomic composition and evolution of *T. luwenshuni* str. Cheeloo. (A) Genome size and G+C content distribution of *T. luwenshuni* str. Cheeloo and the other four *Theileria* species with published complete genome sequences. Different *Theileria* species were indicated with different colors. (B) Bird’s eye view of the assembled genome of *T. luwenshuni* str. Cheeloo showing summary statistics. From the outer circle to the inner circle, six types of information: contig length, genes density, gene annotation (colors imply the KEGG of genes, A09100 Metabolism; A09120 Genetic Information Processing; A09130 Environmental Information Processing; A09140 Cellular Processes; A09150 Organismal Systems; A09160 Human Diseases; A09180 Brite Hierarchies; A09190 Not Included in Pathway or Brite), DNA sequencing data coverage, G+C skew value, and G+C content are labeled. (C) The maximum likelihood phylogenetic tree of *Theileria* species. The tree was inferred by Raxml based on 1,149 single-copy orthologs identified by orthofinder. A total of 1,000 alternative runs were used to calculate support values. Babesia microti and Toxoplasma gondii were two outgroup species to help root the tree. Different *Theileria* species were indicated with different colors. (D) An ultrametric tree of five *Theileria* species, with Babesia microti str. RI and Plasmodium ovale str. PocGH01 as the tree root. Numbers below the branches indicate gene family expansions/contractions, and the numbers above the branches show gene gains/losses.

The genome of *T. luwenshuni* str. Cheeloo was approximately 7.61 Mb in size with 94.47% gene integrity, which was smallest in comparison to those of the other four identified *Theileria* species (Table 1), including *T. annulata* str. Ankara (6), *T. equi* str. WA (26), *T. orientalis* str. Shintoku (5), and *T. parva* str. Muguga (25). After removing bacterial sequences, the estimated genome size of *T. luwenshuni* str. Cheeloo was 7,642,037 bp (Fig. S2), which was comparable to the assembled genome. Total number of contigs in this assembly was 658, the minimum and maximum contig lengths were 504 bp and 363,912 bp, with *L*_50_ and *N*_50_ being 41 and 52,164 bp, respectively (Table 1). MetaEuk was used to predict proteins, and 3,408 putative protein-coding genes were identified in the genome. The G+C content of the *T. luwenshuni* str. Cheeloo genome was 36.55%. There was no obvious association between genome size and G+C content of different *Theileria* species, whose complete genome sequences were available (Fig. 2A). Although the genome size of *T. luwenshuni* str. Cheeloo was smaller than those of *T. annulata* and *T. parva*, it had higher G+C content than the other two *Theileria* species.

**TABLE 1:** Comparison of genome characteristics of the genus *Theileria*

Features	*T. luwenshuni* str. Cheeloo	*T. annulata* str. Ankara	*T. equi* str. WA	*T. orientalis* str. Shintoku	*T. parva* str. Muguga
Size (Mb)	7.61	8.36	11.67	9.01	8.35
G+C content (%)	36.55	32.54	39.48	41.55	34.04
No. of contigs	658	8	12	6	9
Min contig length (bp)	504	5,905	9,001	2,595	13,275
Max contig length (bp)	363,912	2,592,520	3,677,484	2,746,313	2,540,030
*L*_50_	41	2	2	2	2
*N*_50_ (bp)	52,164	1,979,170	2,338,319	2,216,979	1,971,884
No. of protein-coding genes	3408	3796	5329	4002	4051
Avg protein-coding genes length (bp)	1,344	1,605	1,473	1,541	1,467
% of genes with introns	68.1	69	51.3	76.6	70.2
Mean gene length (bp)	1,561	1,775	1,682	1,842	1,878
% coding	60.2	72.8	67.1	68.3	71.1
% G+C composition of exons	41.16	35.71	39.69	46.18	37.74
Gene density (genome size/no. of protein-coding genes)	2233	2202	2190	2251	2060
Busco of protein mode (%)	85.1 (F:6.3, M:8.6)	93.5 (F:2.7, M:3.8)	94.6 (F:3.6, M:1.8)	94.2 (F:2.5, M:3.3)	98.2 (F:0.5, M:1.3)

The description scheme with bird’s eye view demonstrated the basic composition of the *T. luwenshuni* str. Cheeloo genome (Fig. 2B). From the outer to the inner circle, the scheme displayed the contig length, gene density, gene annotation, sequencing data coverage, G+C skew value, and G+C content. The phylogenetic analysis based on the complete genome sequences of *Theileria* species published revealed that *T. luwenshuni* str. Cheeloo occupied a separate evolutionary lineage distinct from the other four *Theileria* species and was genetically close to *T. orientalis* and distant to *T. equi* (Fig. 2C). We constructed the ultrametric tree based on the whole-genome sequences of five *Theileria* species, Babesia microti str. RI, and Plasmodium ovale str. PocGH01 (Fig. 2D). Based on the 233 million years ago (MYA) of median divergence time between *P. ovale* str. PocGH01 and B. microti str. RI, the divergence time between *T. luwenshuni* str. Cheeloo and *T. orientalis* str. Shintoku was about 21 million years ago, which range from 1.8 to 56.7 million years with different calibration points. Notably, *T. luwenshuni* possessed the fewest gene gains and gene family expansion among all the five species, followed by its genetically close species *T. orientalis*. But it had the second most gene losses and gene family contraction just after *T. equi*, which was genetically most distant to *T. luwenshuni* str. Cheeloo. Considering the potential poor spaces in assembly, we conducted the genome synteny analysis between *T. luwenshuni* str. Cheeloo and *T. orientalis*, which are genetically closest to each other. The poor spaces were discovered at the ends of both genomes (Fig. S3). Notably, the difference in many regions of the whole genomes was obvious between the two *Theileria* species. This finding indicates the small size of the *T. luwenshuni* str. Cheeloo genome is unlikely caused by any gene missing.

To better illustrate the evolutionary relationships between *T. luwenshuni* str. Cheeloo and other *Theileria* species, we constructed a phylogenetic tree based on 145 *Theileria* 18S rRNA genes available in GenBank (Fig. S4). *T. luwenshuni* str. Cheeloo was on the same lineage with some unclassified *Theileria* species as well as *T. luwenshuni*. They were clustered with *Theileria* species identified mostly in small ruminants, including *Theileria ovis*, *Theileria capreoli*, and *Theileria cervi*, hosts of which are sheep and other wild ruminants, including water deer, horse deer, reindeer, Odocoileus virginianus, etc. (27–30).

### Genome annotation and characterization of *T. luwenshuni* str. Cheeloo in comparison to other *Theileria* species.

**FIG 3:**
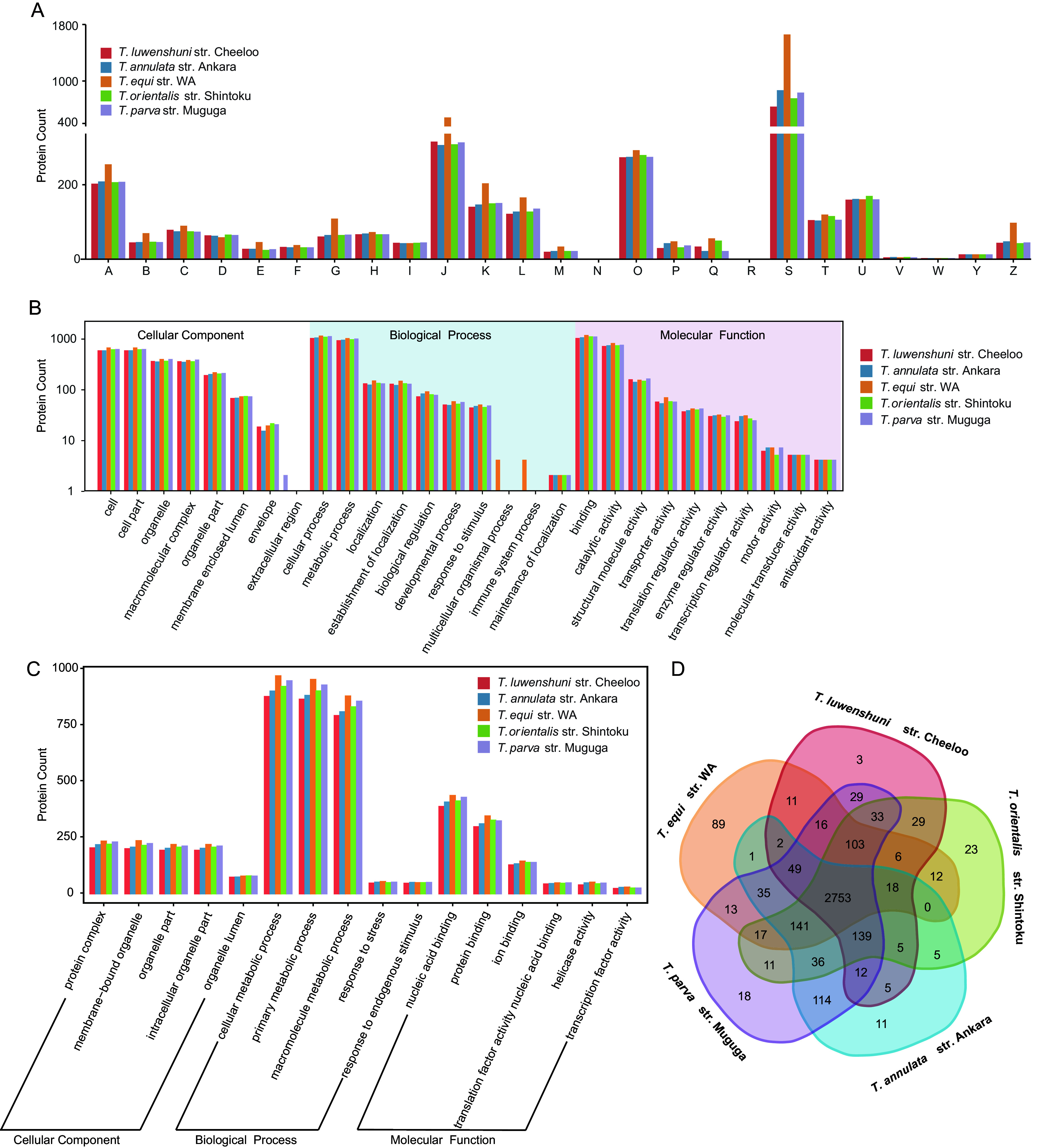
Genome annotation and characterization of *T. luwenshuni* str. Cheeloo in comparison to other *Theileria* species. (A) KOG analysis of all predicted genes. The *x* axis indicates KOG categories and the *y* axis indicates the number of proteins. The *x* axis A to W represent: A, RNA processing and modification; B, chromatin structure and dynamics; C, energy production and conversion; D, cell cycle control, cell division, chromosome partitioning; E, amino acid transport and metabolism; F, nucleotide transport and metabolism; G, carbohydrate transport and metabolism; H, coenzyme transport and metabolism; I, lipid transport and metabolism; J, translation, ribosomal structure and biogenesis; K, transcription; L, replication, recombination and repair; M, cell wall/membrane/envelope biogenesis; N, cell motility; O, posttranslational modification, protein turnover, chaperones; P, inorganic ion transport and metabolism; Q, secondary metabolites biosynthesis, transport and catabolism; R, general function prediction only; S, function unknown; T, signal transduction mechanisms; U, intracellular trafficking, secretion, and vesicular transport; V, defense mechanisms; W, extracellular structures. Y and Z represent nuclear structure and cytoskeleton, respectively. (B) Gene ontology (GO) terms assignment for proteins from five *Theileria* species at level 2. Results are summarized into three categories of cellular component, molecular function, and biological process. (C) GO terms with differential protein counts at level 3. (D) Venn diagram showing number of orthogroups found in different *Theileria* species.

To characterize the genome of *T. luwenshuni* str. Cheeloo and compare the differences in protein-coding genes, we annotated the genes using three different approaches respectively, based on Eukaryote Orthologs (KOG), Gene Ontology (GO), and Kyoto Encyclopedia of Genes and Genomes (KEGG). The functional categories of coding proteins of each *Theileria* species based on KOG annotation are listed in Table S2 and visualized in the bar graphs (Fig. 3A). Overall, the proteins with unknown function had highest counts among all the five species, accounting for 18.72%, 22.92%, 31.13%, 18.89%, and 20.66% of total encoded proteins of *T. luwenshuni* str. Cheeloo, *T. annulata* str. Ankara, *T. equi* str. WA, *T. orientalis* str. Shintoku, and *T. parva* str. Muguga, respectively. Among the protein with known function, the top five categories included “RNA processing and modification,” “translation, ribosomal structure, and biogenesis,” “posttranslational modification, protein turnover, chaperones,” and “transcription” followed by “intracellular trafficking, secretion, and vesicular transport,” “signal transduction mechanisms,” and “replication, recombination, and repair.” Notably, the protein count of each category was always comparable between *T. luwenshuni* str. Cheeloo and *T. orientalis* str. Shintoku (Fig. 3A), though there were remarkable differences in genome size (7.61 Mb versus 9.01 Mb) and in protein count (3,408 versus 4,002) (Table 1).

For further GO annotation, considering that the export format of eggNOG did not meet the requirements, InterProScan was chosen to perform the structural domain similarity comparison between *T. luwenshuni* str. Cheeloo and the other four species. We initially summarized GO categories at level 2, involving Cellular Component, Biological Process, and Molecular Function (Fig. 3B). In the Cellular Component category, the protein counts involving cell, cell part, organelle, macromolecular complex, organelle part, membrane enclosed lumen, and envelope of *T. luwenshuni* str. Cheeloo were comparable to the other four *Theileria* species. The extracellular region was only available for *T. parva* str. Muguga, but not for the other four *Theileria* species. There was no significant difference in protein counts regarding the cellular process, metabolic process, localization, establishment of localization, biological regulation, developmental process, response to stimulus, and maintenance of localization section in the Biological Process category, between *T. luwenshuni* str. Cheeloo and the other four *Theileria*. Only *T. equi* had proteins involving multicellular organismal process and immune system process. All the contents under the Molecular Function category, including binding, catalytic activity, structural molecule activity, transporter activity, translation regulator activity, enzyme regulator activity, transcription regulator activity, motor activity, molecular transducer activity, and antioxidant activity, were also similar among *T. luwenshuni* str. Cheeloo and the other four *Theileria* species.

Furthermore, a bar graph was created to clarify the differences in more detail between *T. luwenshuni* str. Cheeloo and the other four species at level 3 (Fig. 3C). In the Cellular Component category, the protein complex, membrane-bound organelle part, organelle part, and intracellular organelle part in *T. luwenshuni* str. Cheeloo were lower than the other four *Theileria* species, with *T. equi* str. WA being the highest. In the Biological Process category, the cellular metabolic process, primary metabolic process, and macromolecule metabolic process sections in *T. luwenshuni* str. Cheeloo were much lower than the other four *Theileria* species. Regarding the response to stress and endogenous stimulus, *T. luwenshuni* str. Cheeloo did not significantly differ from the other four *Theileria* species. The annotation results under the Molecular Function category, including nucleic acid binding, protein binding, ion binding, translation factor activity nucleic acid binding, and transcription factor activity, were lower for *T. luwenshuni* str. Cheeloo than for the other four species.

Orthogroups of *Theileria* species are presented in the Venn diagram (Fig. 3D). All the five species in the genus *Theileria* shared 2,753 orthogroups in common. *T. luwenshuni* str. Cheeloo processed three unique orthogroups with unknown functions and shared 29 orthogroups with its most closely related *T. orientalis* str. Shintoku, which were absent in *T. equi* str. WA, *T. parva* str. Muguga, and *T. annulata* str. Ankara. In addition, *T. luwenshuni* str. Cheeloo shared 29, 11, and 5 orthogroups with *T. parva* str. Muguga, *T. equi* str. WA, and *T. annulata* str. Ankara, respectively. Compared with other members of the genus *Theileria*, 141 orthogroups in the other four species were absent in *T. luwenshuni* str. Cheeloo.

### Absence of the c-Myb in *T. luwenshuni* str. Cheeloo.

Sustained activation of NF-κB, which saves cells from spontaneous apoptosis, can activate the expression of target genes (31). Intracellular protozoans have evolved a plethora of mechanisms to ensure their dissemination and escape hostile host responses. *Theileria* is the only eukaryote known to induce uncontrolled proliferation of host cells. The survival or apoptosis of *Theileria*-transformed leukocytes is strictly dependent on NF-κB activity (32). c-Myb is present at the end of the NF-κB signaling pathway and is a DNA-binding transcription factor that has functions in apoptosis, proliferation and differentiation (33, 34). In this study, we found that *T. luwenshuni* str. Cheeloo lacked c-Myb by KEGG annotation, which is presented in all other four *Theileria* species. We designed four sets of primers based on the c-Myb in other *Theileria* species (Table S1), none of which could be amplified from the original samples.

### Prevalence and evolution of *T. luwenshuni* in goats.

**FIG 4:**
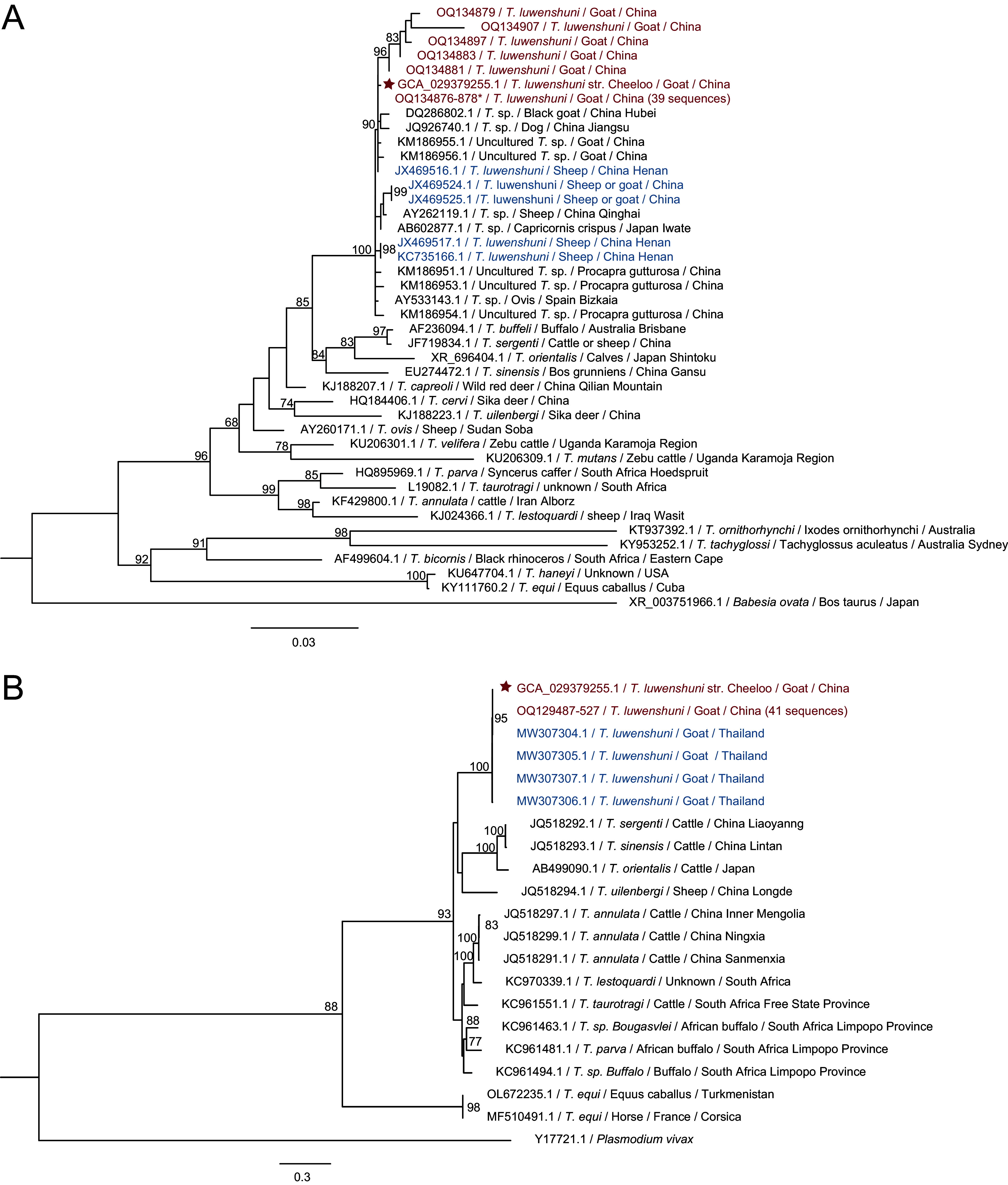
Phylogenetic analysis of *T. luwenshuni* str. Cheeloo based on the full-length of the 18S rRNA gene and the partial *cox1* gene. (A) Phylogenetic tree of *Theileria* based on 18S rRNA gene sequences. (B) Phylogenetic tree of *Theileria* based on *cox1* gene sequences. The sequences obtained in this study are highlighted in red. The stars in panels A and B indicate the sequences from the assembled genome of *T. luwenshuni* str. Cheeloo. The asterisk (*) indicates the 18S rRNA gene sequences obtained in this study with GenBank accession numbers of OQ134880, OQ134882, OQ134884 to OQ134896, OQ134898 to OQ134906, and OQ134908 to OQ134919.

We tested *T. luwenshuni* str. Cheeloo in blood samples from 54 goats of three flocks at Shandong Province of eastern China (see Fig. S1 in the supplemental material) by PCR assays using specific primers targeting 18S rRNA gene (1,745 bp) and the partial cytochrome oxidase subunit 1 (*cox1*) gene (602 bp) of *T. luwenshuni* str. Cheeloo (Table S1) followed by Sanger sequencing. The overall positive rate was up to 81.5% (95% CI, 71.1% to 91.8%). The 18S rRNA genes sequences of *Theileria* detected in the goats of this study (GenBank accession no. OQ134876 to OQ134919) showed an average similarity of over 99.8% with each other. The phylogenetic analysis based on the 18S rRNA genes revealed that all the sequences together with the 18S rRNA gene sequence of *T. luwenshuni* str. Cheeloo were clustered in a separate lineage of the same clade with *T. luwenshuni* and unclassified *Theileria* species in ruminants from China, Japan, and Spain (Fig. 4A). The *cox1* gene sequence (602 bp) amplified from 41 goat samples (GenBank accession no. OQ129487 to OQ129527) in this study had 99.8% to 100% identity to each other and to the corresponding region of *T. luwenshuni* str. Cheeloo. The phylogenetic tree based on the *cox1* gene indicated that *T. luwenshuni* detected in the goats and *T. luwenshuni* str. Cheeloo were clustered with *T. luwenshuni* (GenBank accession no. MW307304.1) detected in goats from Thailand (35) in the same clade, further proving *T. luwenshuni* str. Cheeloo as a *T. luwenshuni* species (Fig. 4B).

## DISCUSSION

In this study, we initially detected *T. luwenshuni* by PCR in goats during a survey on tick-borne agents, observed by microscopic examination of Wright-Giemsa staining slides, and confirmed by FISH using a specific probe. After directed metagenomic next-generation sequencing of an infected goat blood sample, we then assembled a complete genome sequence of *Theileria*, designated *T. luwenshuni* str. Cheeloo. This is the genome of the fifth *Theileria* species obtained in the world. Whole-genome assembly of intracellular parasites has usually been prohibited by the DNA presence of host cells. Considering *T. luwenshuni* is only observed in erythrocytes by Wright-Giemsa staining and FISH, we first separated erythrocytes from the infected goat blood, and then lysed them for maximum removal of goat DNA. After metagenomic sequencing, the remaining goat genomic sequences were further discarded, and the *T. luwenshuni* str. Cheeloo genome was successfully assembled from the infected goat. The gene integrity is up to 94.47%. The phylogenetic analysis based on the *Theileria* genome sequences, and the comparative analyses of genomic characteristics revealed that *T. luwenshuni* str. Cheeloo is distinct from the other four *Theileria* species, and more closely related to *T. orientalis*.

A morphological test has been traditionally applied for diagnosing protozoan infections, but sometimes is not specific. FISH tests using probes have been used for specific identification of protozoans such as *Plasmodium* and *Babesia* (36, 37). In this study, we developed a specific FISH assay for *T. luwenshuni.* Furthermore, we simultaneously stained the nucleus using DAPI to verify the morphological features of *Theileria* through fluorescence microscopy. The morphology of *T. luwenshuni* str. Cheeloo within the erythrocytes is diverse, with various shapes such as rod-shaped, ovoid, and pear-shaped. Previous studies have indicated that upon entry into the host, some *Theileria* species initially undergo schizont proliferation in host leukocytes, forming multinucleated schizonts, which further develop into a uninucleate merozoite stage. The merozoites are released from the leukocytes and invade the erythrocytes, subsequently becoming piroplasms (38–41). To date, schizonts have been directly observed in leukocytes of bovines infected with *T. annulata*, *T. parva*, and *T. orientalis* (41, 42). In our study, no schizont is visualized in the leukocytes of goats infected with *T. luwenshuni* str. Cheeloo by either Wright-Giemsa staining or FISH. We are uncertain whether *T. luwenshuni* str. Cheeloo has an intraleukocyte schizont stage in the peripheral blood, as previously observed in other *Theileria* species infecting bovines (43).

The genomic composition of *T. luwenshuni* str. Cheeloo has some distinct features compared with the other four *Theileria* species. The genome size of *T. luwenshuni* str. Cheeloo is smallest (around 7.61 Mb) among the *Theileria* genomes assembled until now. *T. luwenshuni* str. Cheeloo shares more genes with both *T. orientalis* and *T. parva* that are not present in the other two *Theileria* species. *T. luwenshuni* and *T. orientalis* are closer in evolutionary distance and time than the other three *Theileria* species, which may explain why they share the more protein-coding genes. *Theileria luwenshuni* str. Cheeloo processes fewer protein-coding genes than the other four *Theileria* species in the Cellular Component classification at level 3 of the GO annotation, involving membrane-bound organelle, organelle, and intracellular organelle. These data will undoubtedly improve our understanding of the gene function of *T. luwenshuni* str. Cheeloo. Notably, KEGG annotation reveals the absence of c-Myb in *T. luwenshuni* str. Cheeloo, indicating it might have different mechanisms in comparison with other *Theileria* species in apoptosis, proliferation, and differentiation (33, 34).

The infection rate of *T. luwenshuni* is up to 81.5% in goats from Shandong Province of eastern China, which is higher than those detected in Chinese *Cervidae* from northwestern China, and small ruminant from central and northwestern China (9, 10, 44). The reason for the high prevalence in goats of Shandong Province might be due to sampling bias. On the other hand, the finding might indicate the higher infectivity of *T. luwenshuni* str. Cheeloo, which needs further experimental studies to prove this hypothesis. We further did the phylogenetic analyses based on both 18S rRNA and *cox1* genes of *Theileria* by including more sequences from different animal hosts and found that *T. luwenshuni* from different hosts and geographic locations were always clustered in the same clade that is more genetically close to those detected from ruminants. This finding is consistent with the results demonstrated by some previous studies (45, 46), suggesting a low genetic diversity of *T. luwenshuni*.

## MATERIALS AND METHODS

### Sample collection and DNA extraction.

In the present study, goat blood samples were collected in Shandong Province of eastern China in September 2021 and July 2022. A total of 54 EDTA blood samples were collected from three flocks of goats. Eleven samples were collected from one flock of goats for the first collection and 43 blood samples (six of the blood samples were from the same site as the first collection) were collected from three flocks of goats for the second collection (Fig. S1). A High Pure PCR template preparation kit (Roche, Germany) was used for DNA extraction from blood samples according to the manufacturer’s instructions.

### PCR amplification and sequencing.

A PCR assay specific for the 18S rRNA gene of *Theileria* and *Babesia* was conducted (Table S1) to screen the goat blood samples of the first flock, as previously described (47). The primers specific for the c-Myb gene were designed according to the conserved regions of the four *Theileria* species with published genomes (Table S1) and used for amplification of the gene from goat blood samples. All 54 blood samples were amplified for 18S rRNA and *cox1* gene by PCR assays (Table S1). All amplicons were confirmed by Sanger sequencing.

### Wright-Giemsa staining.

Blood smears were prepared on the day of collection, followed by fixation with methanol for 15 to 20 min and drying at room temperature. After the staining was completed, the slides were tilted at 45 degrees, rinsed slowly with water of pH close to neutral, washed of the excess staining solution, and then dried and placed under the microscope for observation.

### Fluorescence *in situ* hybridization.

We used fluorescence *in situ* hybridization (FISH) to observe *T. luwenshuni* on blood smears. FISH probes were designed using 18S rRNA sequences obtained from the whole genome of the *T. luwenshuni* str. Cheeloo (Table S3) labeled with FAM. Blood smears that had been fixed in methanol and stained with Wright-Giemsa were first soaked in 70% ethanol, incubated overnight at 4°C, and then the ethanol was aspirated. The pooled FISH probes were resuspended in a final concentration of 12.5 μM in RNase-free storage buffer and protected from light by storage at −20°C. FISH was performed on the prepared blood smear with a commercial kit according to the manufacturer’s instructions (Biosearch Technologies, California, America). Finally, a drop of fluorescent antibody blocker containing DAPI was placed on the slide, then the slide was covered with a coverslip, incubated for 2 h at room temperature, and observed under a fluorescence microscope (DAPI and FITC channels).

### Enrichment of *Theileria* for genomic sequencing.

Erythrocytes from *Theileria*-infected goats were separated by gradient centrifugation using cell separation solution (Eppendorf, Hamburg, Germany) for 20 min at 200 × *g* at 4°C. Then, four times volume of precooled (4°C) erythrocyte lysis buffer (Solarbio, Beijing, China) was added to the isolated erythrocytes by gentle pipetting to ensure adequate mixing. After placing at 4°C for 10 min, the lysis solution was centrifuged at 350 × *g* for 10 min to remove residual blood cells. Finally, the supernatant was centrifuged at 20,000 × *g* at 4°C for 30 min. The pooled *Theileria* was resuspended for DNA extraction using the High Pure PCR template preparation kit (Roche, Mannheim, Germany).

### Genome assembly and quality assessment.

A sequencing library was then constructed using the AxyPrep Mag PCR clean up kit for MGI Tech Co., Ltd. Then the sequencing library was prepared according to the Whole Genome Sequencing Library Preparation Protocol (DNBSEQ). The paired-end (PE) libraries were sequenced with a read length of 2 × 150 bp on a DNBseq-T7 platform at Grandomics Gene Technology Beijing Co. Ltd. (Beijing, China). High-quality reads were aligned to the goat (*Capra hircus*) genome (GenBank assembly accession no. GCA_001704415.2) using bowtie2 v2.4.1 (48) with default parameters, to remove the genome sequence of the host. Both members of a read pair were discarded if one read matched the *C. hircus* genome by using Samtools v1.9 (49) with parameters -f 12 with 4.1% of reads retained. The remaining reads were sent to SPAdes v3.15.2 (50) with parameters -meta to assemble the *Theileria* genome. Binning and genome reconstruction were accomplished by MetaBAT2 v2:2.15 (51). EukCC v2.1.0 (52) was used to evaluate the completeness of the assembly. The quality of genome assembly was evaluated by QUAST (53). K-mer counting was implemented by Jellyfish (54). The genomescope2 (55) was then used to estimate genome size.

### Phylogenetic analyses.

The sequences of published complete genome sequences of *Theileria* species, including *T. annulata* str. Ankara, *T. equi* str. WA, *T. orientalis* str. Shintoku, and *T. parva* str. Muguga, were downloaded from NCBI. B. microti and T. gondii were selected as outgroups.

The required 18S rRNA and *cox1* gene sequences were also downloaded from NCBI, and multiple sequence alignment (MSA) was then performed using MEGA11. Finally, the maximum likelihood method was used to construct phylogenetic trees.

### Divergence time estimation.

Gene families were identified using OrthoFinder v2.5.4 (56) with default parameters among 12 *Theileria* species and two outgroup species. Proteins from single-copy gene families (*n* = 1,176) were used for subsequent phylogenetic analysis. The protein sequences were aligned using MUSCLE v3.8.1551 (57). Gblocks v0.91b (58) was used to select conserved blocks from aligned sequences. Raxml v8.2.12 (59) was used to generate a phylogenomic tree. The phylogenomic tree used for divergence time predict was built with same pipeline as described above. The divergence time was estimated by r8s (60) using the divergence time of B. microti str. RI and *P. ovale* str. PocGH01 from the TimeTree website (61). CAFE (62) was used to identify gene family expansion and contraction.

### Protein prediction and annotation.

Protein prediction was accomplished by metaEuk v5.34 (63) with default parameters. Busco v4.1.2 with protein mode (64) was used to investigate the accuracy of gene annotation. G+C content and G+C skew value were calculated by GCcalc v1.0.0. InterProScan v5.57-90.0 (65) was used to obtain protein function classification. GO annotations analyzing and plotting were achieved by WEGO v2.0 (66). The GO annotation at both level 2 and level 3 were used to characterize protein-coding genes. KEGG annotation was applied by KofamScan v1.3.0 (67).
