# Supplementary material for: Genomic Characterization of Theileria luwenshuni Strain Cheeloo
Source: Microbiol Spectr. 2023 Jun 1;11(4):e00301-23. doi: 10.1128/spectrum.00301-23 (PMC10434005; doi:10.1128/spectrum.00301-23)
Supplement: Supplemental file 1 — Supplemental material. Download spectrum.00301-23-s0001.pdf, PDF file, 4.3 MB [file spectrum.00301-23-s0001.pdf]

SUPPLEMENTARY MATERIALS

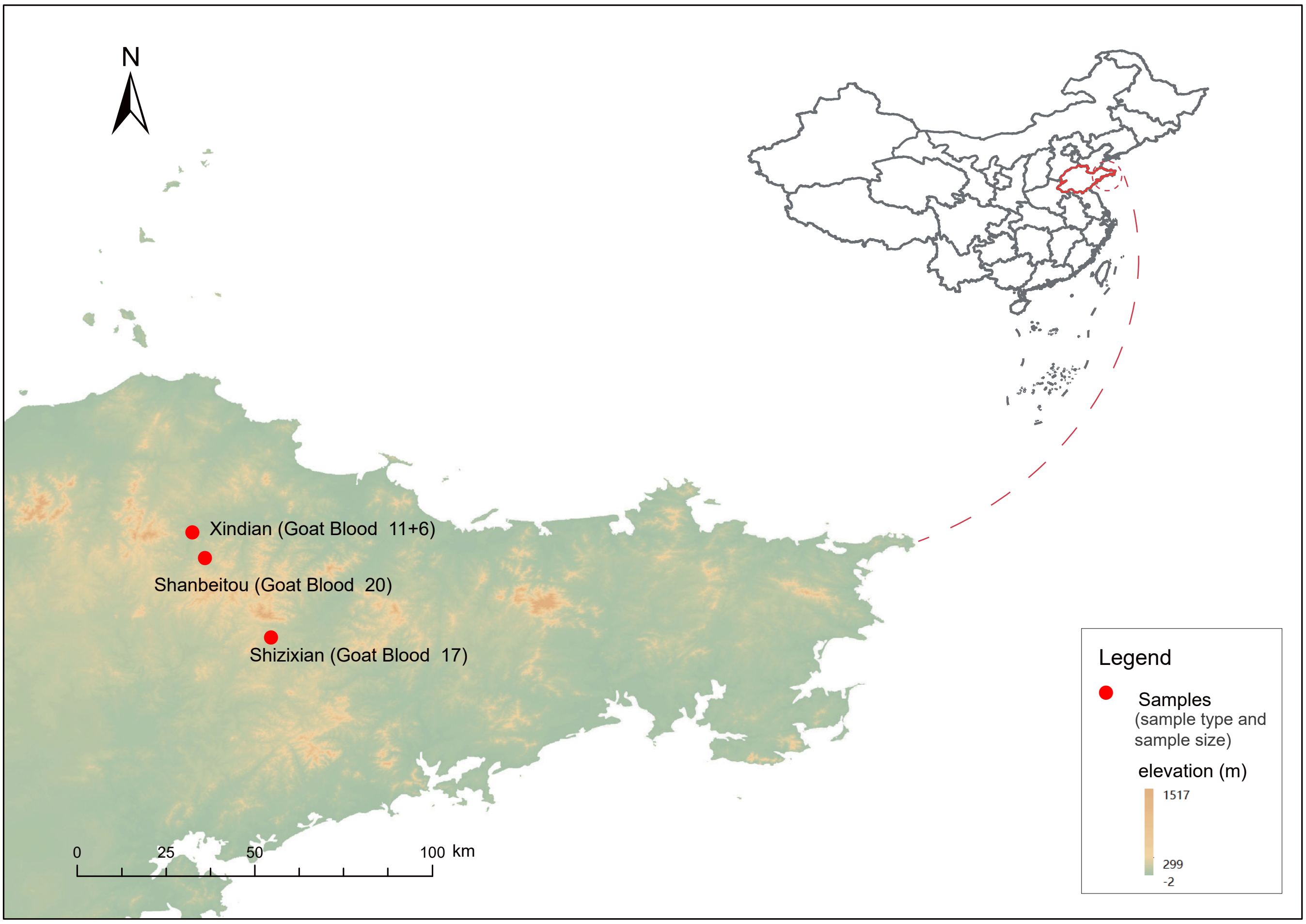

FIG S1 Distribution of the locations used to collect goat blood samples in Shandong Province. The latitude and longitude of the sampling sites are indicated in the figure by the red solid circles. The sample size is shown at each sampling point.

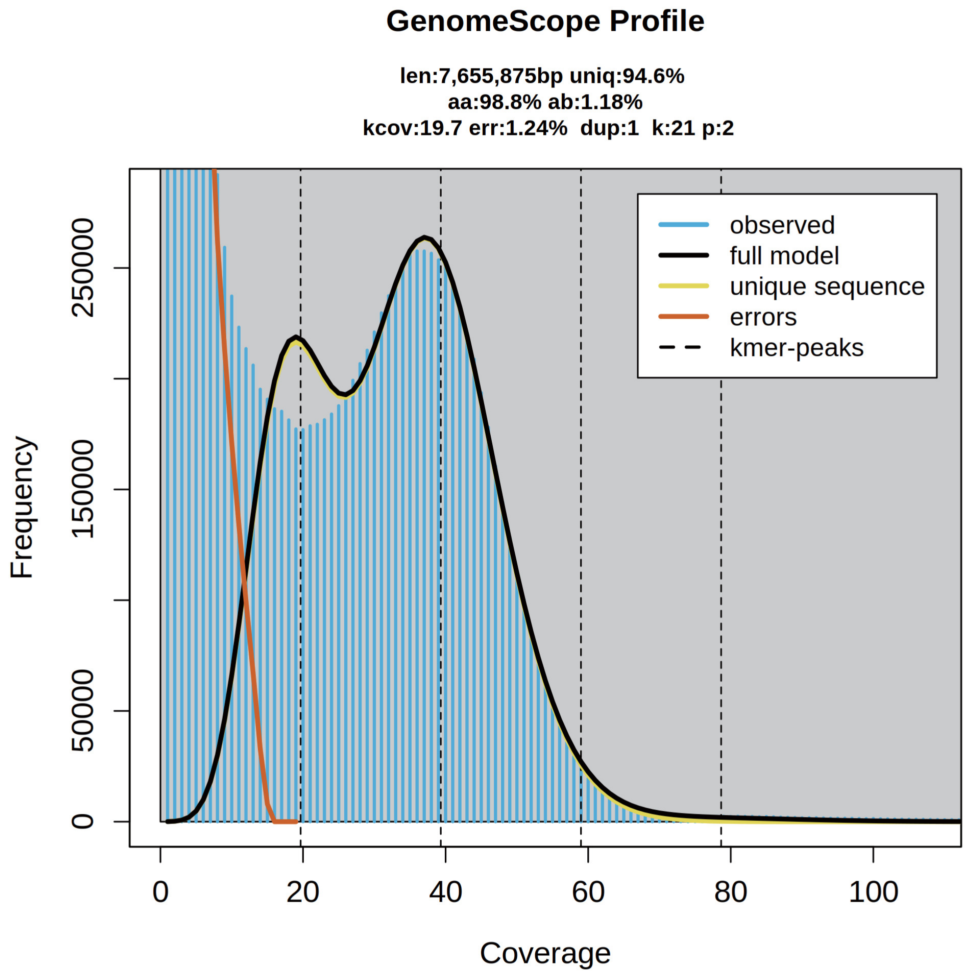

FIG S2 Genome size estimation of *T. luwenshuni* str. Cheeloo. K-mer spectra output is generated from non-host and non-bacteria sequencing data using GenomeScope2.0.

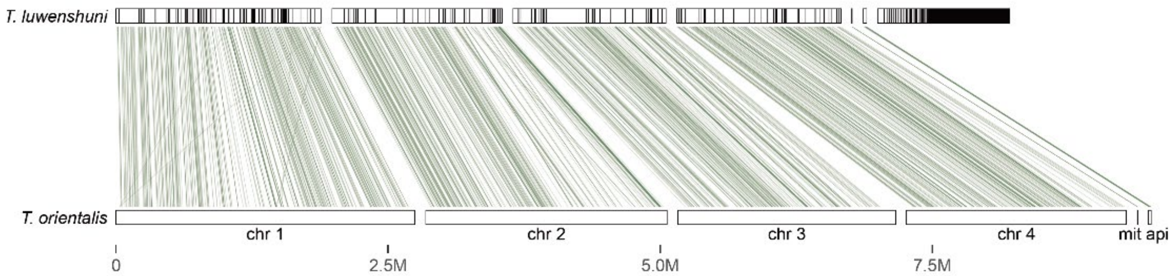

FIG S3 The genome synteny analysis between *T. luwenshuni* str. Cheeloo and *T. orientalis*

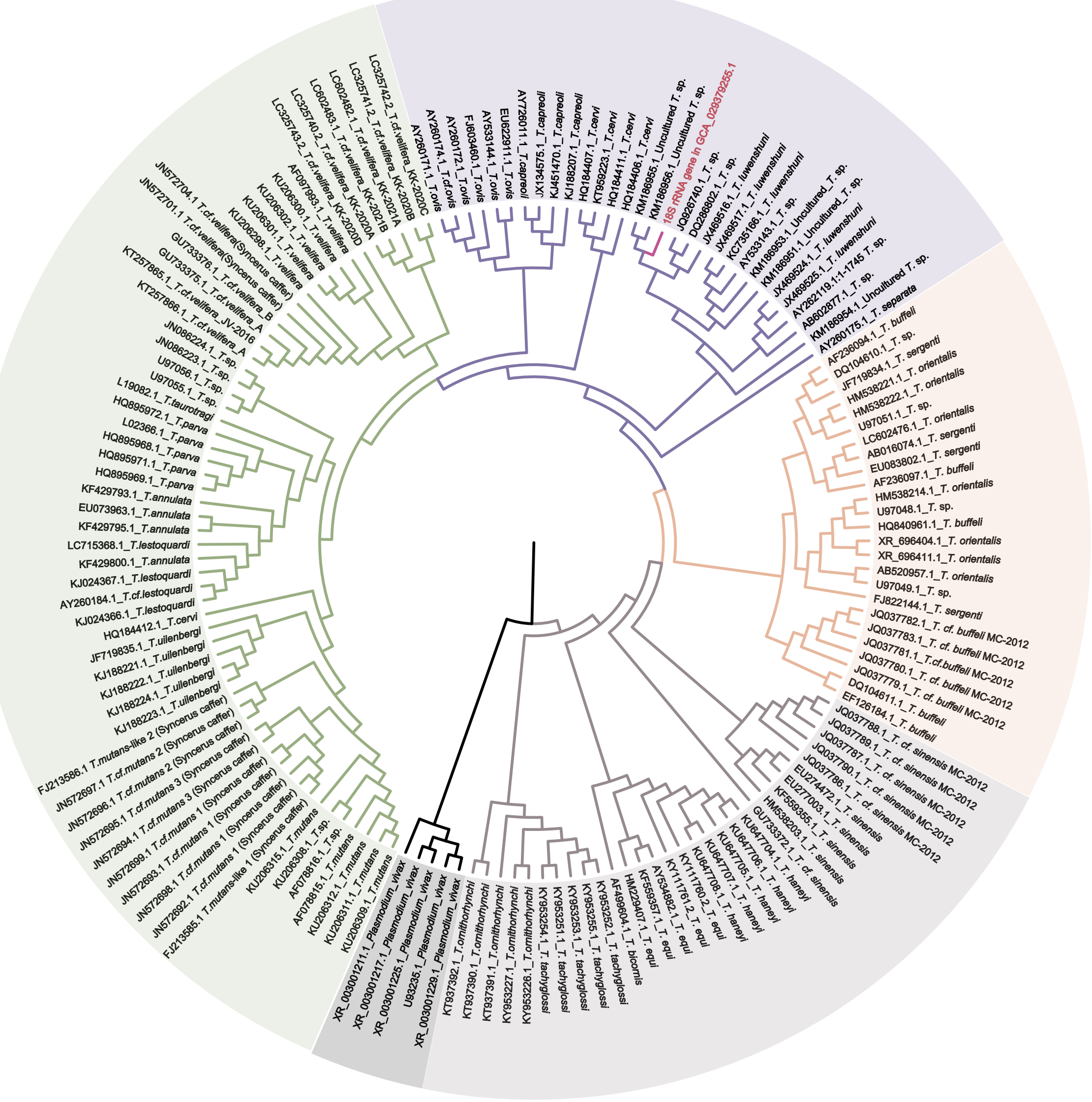

FIG S4 Phylogenetic tree of *Theileria* based on 18S rRNA gene sequences. The phylogenetic tree was constructed using 956-bp 18S rRNA gene sequences of *Theileria* species, with 18S rRNA gene sequences of *Plasmodium vivax* as outgroups. If more than five sequences of a *Theileria* species available in GenBank, only five sequences representative different locations were included in the analysis. If five or fewer sequences of a species were available in GenBank, all the available sequences were included. As a result, a total of 145 sequences, including one extracted from the genome of *T. luwenshuni* str. Cheeloo, 124 from 50 *Theileria* species and 20 from unclassified *Theileria* species, were included in the analysis. GenBank accession number of each sequences is shown in front of the species name. The different clades in the phylogenetic tree are shaded in different colors. The *Theileria* 18S rRNA sequence obtained from the assembly in this study is shown in red.

Table S1. PCR primers used in this study

Gene target	Primer	Primer sequence (5'-3')	Size(bp)	Annealing temperature(℃)	Reference
Specific primers for 18S rRNA gene of *Babesia* and *Theileria*	piro A	AATTACCCAATCCTGACACAG	424	58	1
	piro B	TAAATACGAATGCCCCCAA			
c-Myb#	420Ta. F	TGAAATGCAACCACCAAAGGGAT	1418	56	This study
	420Ta. R	CCTCCGATGATCTTCTTTCCACC			
	420Te.F	TGAACAACAACCACCTAAAGGGT	1418	56	This study
	420Te.R	CGTTAGAAGATGCTCTCTCCCGT			
	420To.F	TGAGATACAGCCGCCAAAAGGAT	1418	61	This study
	420To.R	CACTCGAGGCGAGCCTCTCCCGG			
	420Tp.F	TGAAATGCAACCTCCGAAGGGAT	1418	56	This study
	420Tp.R	CCTCCGATGATCTTCTTTCCACC			
Full-length of 18S rRNA gene of *Theileria*	Primer A	AACCTGGTTGATCCTGCCAGT	1745	56	2, 3
	Primer B	GATCCTTCTGCAGGTTCACCTAC			
*cox1**	*cox1*.25-F1	CTTGCCGCTGGTATAGCTAGTAC	602	54	This study
	cox1.626-R1	CCTATAACAAAGTTAACAACAAACAAT			

*indicates that the *cox1* gene is a gene sequence from *Theileria luwenshuni* that has been published on NCBI (National Center for Biotechnology Information).
#420Ta. F/R was designed based on the sequence of *Theileria annulata*, 420Te.F/R was designed based on *Theileria equi*, 420To.F/R was designed based on *Theileria orientalis*, 420Tp.F/R was designed based on *Theileria parva*.

Primer References:

1. Jiang JF, Zheng YC, Jiang RR, Li H, Huo QB, Jiang BG, Sun Y, Jia N, Wang YW, Ma L, Liu HB, Chu YL, Ni XB, Liu K, Song YD, Yao NN, Wang H, Sun T, Cao WC. 2015. Epidemiological, clinical, and laboratory characteristics of 48 cases of "Babesia venatorum" infection in China: a descriptive study. Lancet Infect Dis 15:196-203.

2. Medlin L, Elwood HJ, Stickel S, Sogin ML. 1988. The characterization of enzymatically amplified eukaryotic 16S-like rRNA-coding regions. Gene 71:491-9.

3. Yang L, Wang J-H, Upadhyay A, Zhao J-G, Huang L-Y, Liao C-H, Han Q. 2022. Identification of Theileria spp. and investigation of hematological profiles of their infections in goats in Hainan Island, China. Parasite 29.

Table S2. Functional clusters of protein-coding genes based on Eukaryote Orthologs Groups annotation of different *Theileria* species

Functional category	*T.luwenshuni-like* str.cheeloo	*T.annulata* str.Ankara	*T.equi* str.WA	*T.orientalis* str.Shintoku	*T.parva* str.Muguga
	Number of genes				
RNA processing and modification	203	209	255	207	208
Chromatin structure and dynamics	45	46	70	47	46
Energy production and conversion	79	75	90	75	74
Cell cycle control, cell division, chromosome partitioning	64	63	59	66	65
Amino acid transport and metabolism	28	28	46	25	27
Nucleotide transport and metabolism	33	32	38	32	32
Carbohydrate transport and metabolism	61	65	109	65	66
Coenzyme transport and metabolism	67	69	73	67	67
Lipid transport and metabolism	44	43	43	44	45
Translation, ribosomal structure and biogenesis	316	307	485	309	314
Transcription	141	147	204	150	151
Replication, recombination and repair	122	128	166	128	136
Cell wall/membrane/envelope biogenesis	20	22	34	22	22
Cell motility	1	1	1	1	1
Posttranslational modification, protein turnover, chaperones	274	275	293	280	275
Inorganic ion transport and metabolism	30	43	48	32	37
Secondary metabolites biosynthesis, transport and catabolism	34	22	56	50	22
General function prediction only	0	0	0	0	0
Function unknown	638	870	1659	756	837
Signal transduction mechanisms	105	104	120	116	106
Intracellular trafficking, secretion, and vesicular transport	160	162	161	170	161
Defense mechanisms	5	6	5	6	5
Extracellular structures	3	3	3	3	3
Nuclear structure	13	13	13	13	13
Cytoskeleton	44	48	98	43	45

Table S3 The probes used in fluorescence in situ hybridization (FISH) assay

Probe Sequence (5' to 3')	Probe sequence name
TATGACTACTGGCAGGATCA	Theileria 18S-1
ACTTAGACATGCATGGCTTA	Theileria 18S-2
TTGTAATGAGCCATTCGCAG	Theileria 18S-3
TAGCACGGTTATCCATGTAA	Theileria 18S-4
TCGAACATGTATTAGCCCTA	Theileria 18S-5
TCTAATAAACGCCACCCGAA	Theileria 18S-6
TTATGAATCACCGTCAACCG	Theileria 18S-7
AAAGCTGCGATTCGCAAGTT	Theileria 18S-8
CGTCAAGCTGATAGGTCAGA	Theileria 18S-9
CGGAATCGAACCCTAATTCC	Theileria 18S-10
CTGTGTCAGGATTGGGTAAT	Theileria 18S-11
GTTATTTCTTGTCACTACCT	Theileria 18S-12
TTACAAGACATTAAGCCCCG	Theileria 18S-13
GTTTAAATTCCCATCATTCC	Theileria 18S-14
TACGCTATTGGAGCTGGAAT	Theileria 18S-15
GCCACAATACACCAACTCAT	Theileria 18S-16
AAGGCAAAAGCCTGCTTTGA	Theileria 18S-17
CCTACTTTATTATTCCATGC	Theileria 18S-18
CCAACTGTTCCTATTAACCA	Theileria 18S-19
GAATTTCACCTCTGACAGTT	Theileria 18S-20
GCAGTAGTTCGTCTTTAACA	Theileria 18S-21
AACATCCTTGGCAAATGCTT	Theileria 18S-22
TACGACGGTATCTGATCGTC	Theileria 18S-23
GTCGGCATAGTTTATGGTTA	Theileria 18S-24
AAACTGACGACCTCCAATCT	Theileria 18S-25
CTCTCAAGGTGCTGAAGGAG	Theileria 18S-26
CCAGAACCCAAAGACTTTGA	Theileria 18S-27
TTCCGTCAATTCCTTTAAGT	Theileria 18S-28
GAGTCAAATTAAGCCGCAGG	Theileria 18S-29
GCTATCAATCTGTCAATCCT	Theileria 18S-30
CCACCACCCAAAGAATCAAG	Theileria 18S-31
ACCAACTAAGAACGGCCATG	Theileria 18S-32
CCTATTTAGCAGGTTAAGGT	Theileria 18S-33
ATCACGGGACAGCAAAAGCT	Theileria 18S-34
CGCAAAGTCCCTCTAAGAAG	Theileria 18S-35
GTTATTGCCTTAAACTTCCT	Theileria 18S-36
AGGACATCTAAGGGCATCAC	Theileria 18S-37
GATGAACGCATCAGTGTAGC	Theileria 18S-38
AAAGATTACCCAGACCTCTC	Theileria 18S-39
CAATAATCGATCCCCATCAC	Theileria 18S-40
CATTCCTCGTTCACGATTAA	Theileria 18S-41
CTGATGACTTGCGCATACTA	Theileria 18S-42
AAAGGGCAGGGACGTAATCT	Theileria 18S-43
GATCACTCGATCGGTAGGAG	Theileria 18S-44
GAAACATCGCGGTCCGAATA	Theileria 18S-45
AAACTTCCCTAGACGTAACC	Theileria 18S-46
TTCTCCTTCCTTTAAGTGAT	Theileria 18S-47
CACCTACGGAAACCTTGTTA	Theileria 18S-48
